# Clinicopathological features of non-cirrhotic portal fibrosis in primary biliary cholangitis: a 15-year experience at a tertiary referral center

**DOI:** 10.3389/fmed.2026.1696121

**Published:** 2026-04-07

**Authors:** Xiuhong Wang, Lina Zhang, Shixuan Liu, Tong Zhang, Tailing Wang, Dingrong Zhong

**Affiliations:** 1 Department of Pathology, China-Japan Friendship Hospital, Beijing, China; 2 Department of Rheumatology and Immunology, Peking University International Hospital, Beijing, China; 3 Department of Pathology, Peking University International Hospital, Beijing, China

**Keywords:** non-cirrhotic portal fibrosis, obliterative portal venopathy, portal hypertension, porto-sinusoidal vascular disease, primary biliary cholangitis

## Abstract

**Background:**

Non-cirrhotic portal fibrosis (NCPF) has been reported to contribute to portal hypertension in primary biliary cholangitis (PBC) preceding the development of cirrhosis. However, the prevalence and clinical significance of NCPF in early PBC remain poorly defined. This study aimed to evaluate the clinicopathological features of NCPF in pre-cirrhotic PBC.

**Methods:**

A retrospective analysis was conducted on 92 biopsy-proven pre-cirrhotic PBC patients from a total of 109 cases collected over 15 years. Clinical/serological characteristics and histological features were compared between the PBC-only and PBC + NCPF groups.

**Results:**

NCPF was identified in 30/92 (32.6%) pre-cirrhotic PBC patients. Specifically, within the subgroup of 71 patients who had early-stage disease (Scheuer stages 1 and 2), NCPF was present in 20 (28.2%) patients. The PBC + NCPF group was significantly older (54.2 ± 8.5 vs. 49.4 ± 11.4 years, *p* = 0.04) and demonstrated elevated alanine aminotransferase (ALT) (102.4 ± 88.9 vs. 69.3 ± 47.1 U/L, *p* = 0.02) and aspartate aminotransferase (AST) levels (103.2 ± 67.5 vs. 70.3 ± 41.0 U/L, *p* = 0.02). Non-significant trends toward elevated alkaline phosphatase (ALP) and IgG levels, along with increased positivity for antinuclear antibody (ANA) and antimitochondrial antibody (AMA), were observed in the PBC + NCPF group. Histological examination revealed typical features of obliterative portal venopathy (OPV) in the PBC + NCPF group, including luminal narrowing and sclerosis of portal vein branches. Immunohistochemistry revealed significantly increased angiogenesis in the PBC + NCPF compared with the PBC-only group.

**Conclusion:**

This study identifies a high prevalence of NCPF in early PBC. Its association with greater biochemical activity and specific vascular histopathological alterations suggests that the presence of NCPF may delineate a more active PBC phenotype, offering potential value for early risk assessment.

## Introduction

Primary biliary cholangitis (PBC) is a progressive autoimmune liver disorder characterized by immune-mediated destruction of intrahepatic bile ducts, potentially leading to fibrosis and cirrhosis (1). While cirrhosis-related complications are well-documented, emerging evidence indicates that portal hypertension and splenomegaly frequently occur in pre-cirrhotic PBC stages (2–6). Recent studies have documented the occurrence of non-cirrhotic portal fibrosis (NCPF) in early PBC (5, 6), a condition characterized by intrahepatic vascular lesions and portal hypertension in the absence of cirrhosis. This clinical paradox underscores critical knowledge gaps regarding early microvascular injury in PBC. Growing evidence implicates that porto-sinusoidal vascular disease (PSVD), which encompasses lesions such as obliterative portal venopathy (OPV) and sinusoidal remodeling, contributes to non-cirrhotic portal hypertension in autoimmune liver diseases (7–10). Nevertheless, the systematic histological evaluation of PSVD subtypes, particularly NCPF, and their clinical relevance in early PBC cohorts remains largely unexplored (5, 10).

Critically, whether NCPF contributes to the heterogeneity of hepatocellular injury, serological profiles, or portal hypertensive manifestations in early PBC requires further investigation. Prior studies have focused primarily on advanced PBC or isolated PSVD, neglecting systematic comparisons between early PBC with and without NCPF. This knowledge gap impedes risk stratification and mechanistic insights into atypical disease progression to portal hypertension in early PBC.

To resolve these uncertainties, this study analyzed 92 biopsy-confirmed pre-cirrhotic PBC patients. The study aimed to (1) evaluate the prevalence of NCPF in PBC using the Asian Pacific Association for the Study of the Liver (APASL) histopathological criteria, (2) compare clinical and serological features between PBC subgroups stratified by NCPF, and (3) delineate characteristic histological lesions in PBC + NCPF patients. The findings of this study provide novel insights into the underrecognized role of microvascular injury in early PBC pathogenesis.

## Methods

### Patients

Biopsy-proven PBC patients were retrospectively enrolled at the China–Japan Friendship Hospital from 2006 to 2021. Among patients referred to the hospital for unknown causes of liver dysfunction, 109 cases were diagnosed with PBC following a comprehensive assessment of clinical and pathological data. All liver specimens were reviewed by pathologists with expertise in hepatopathology. Among the 109 PBC patients, 92 pre-cirrhotic cases were included after excluding those with inadequate specimens, cirrhosis, or overlapping diseases. To minimize misclassification, PBC–AIH overlap was assessed according to the Paris criteria (11, 12), incorporating biochemical/serological parameters (including ALT and IgG) together with histological assessment for interface hepatitis; patients meeting the Paris criteria for PBC–AIH overlap were excluded. All data were anonymized in this retrospective study, and the requirement for informed consent was waived by the Ethics Committee of the China–Japan Friendship Hospital. The study protocol was approved by the Ethics Committee of the China–Japan Friendship Hospital. This study was conducted in accordance with the ethical guidelines of the Declaration of Helsinki.

### Histologic evaluation

All archived Hematoxylin–Eosin, D-PAS, Masson trichrome, and reticulin-stained liver biopsy slides were re-examined, and all histologic findings were documented. PBC diagnosis was based on liver function tests, the presence of serum antimitochondrial antibodies, and bile duct injury in the portal area on liver histology (13). Two experienced liver pathologists (Wang XH and Wang TL) evaluated the biopsy sections and reached a consensus on histologic findings.

NCPF was defined according to the APASL recommendations (7) by meeting at least one of the following criteria: (1) specific histological features such as OPV, nodular regenerative hyperplasia, and incomplete septal fibrosis; (2) non-specific histological features, such as portal tract abnormalities (e.g., aberrant vessels) and architectural disturbance, including irregular distribution of portal tracts and central veins, non-zonal sinusoidal dilation, and mild peri-sinusoidal fibrosis.

Histological staging was performed using the Scheuer staging system, which comprises four stages (14). Stage 1 is characterized by portal inflammation with granulomatous destruction of the bile ducts. Stage 2 is characterized by mild periportal fibrosis and bile duct proliferation. Stage 3 is defined by the presence of fibrous septa or bridging necrosis, and stage 4 by cirrhosis.

### Statistical analysis

SPSS 26.0 (IBM, Armonk, NY, USA) was used for data analysis. Descriptive data were presented as mean ± standard deviation (SD) for continuous variables and as frequencies and percentages (%) for categorical variables. Analysis of variance was used to compare the means of continuous data. Categorical variables were evaluated using the chi-square test or Fisher’s exact test. Trends in the proportion of splenomegaly and NCPF across different Scheuer stages were examined using the Cochran–Armitage trend test. A two-sided *p*-value < 0.05 was considered statistically significant.

## Results

### Patient characteristics

**Figure 1:**
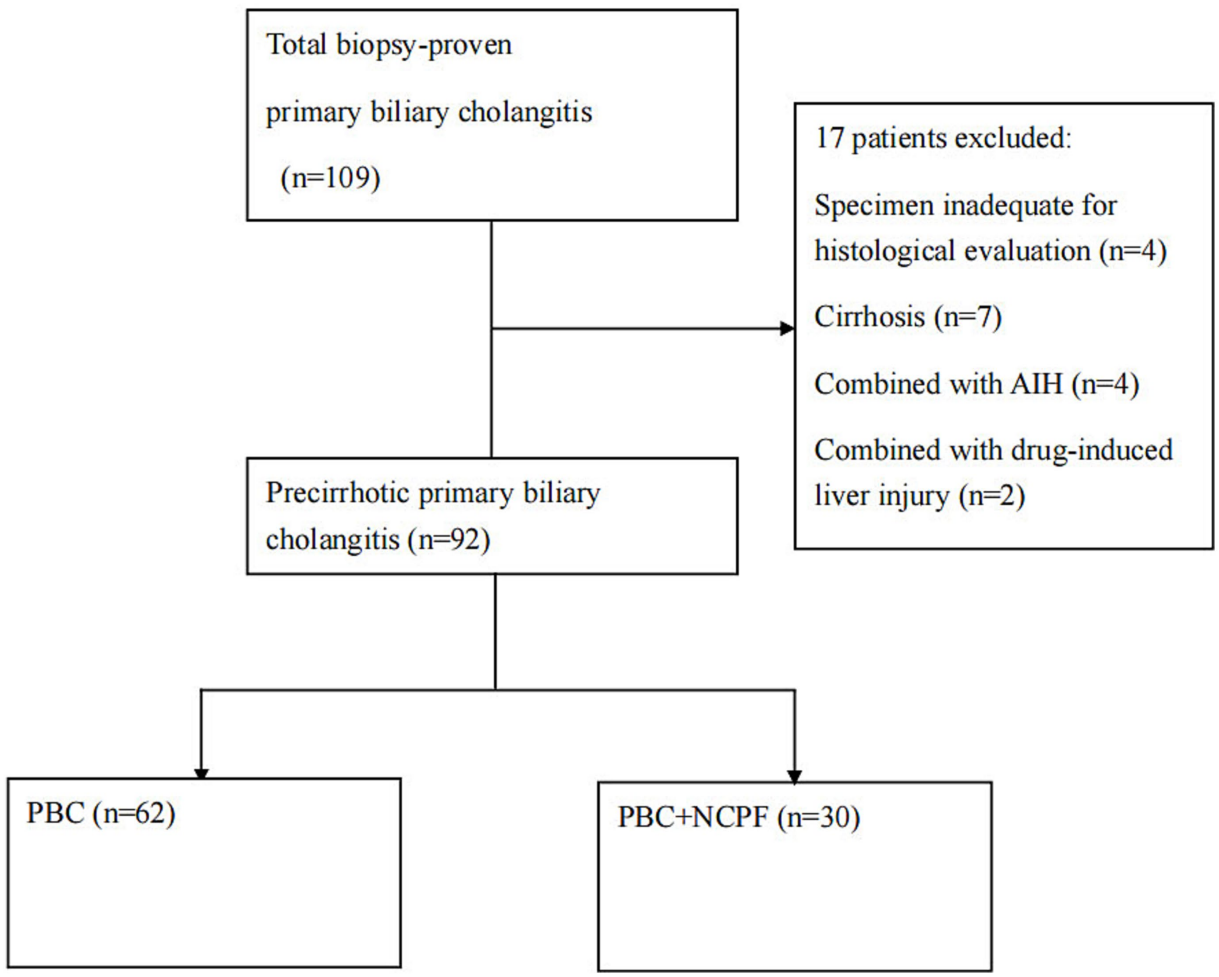
Flowchart of the patient enrolment process. AIH, autoimmune hepatitis; NCPF, non-cirrhotic portal fibrosis; PBC, primary biliary cholangitis.

A total of 109 biopsy-proven PBC patients were initially examined (Figure 1). Ninety-two pre-cirrhotic PBC patients were enrolled after excluding those with inadequate specimens (*n* = 4), cirrhosis (*n* = 7), overlap with drug-induced liver injury (*n* = 2), or autoimmune hepatitis (*n* = 4). There were 85 women and 7 men, with a mean age of 51.0 ± 10.7 years. All liver specimens were reviewed by hepatopathologists. All patients were classified as pre-cirrhotic PBC according to Scheuer’s classification (stages 1–3).

Among the 92 patients with pre-cirrhotic PBC, 30 were identified as having NCPF according to APASL recommendations (7). Specifically, within the subgroup of 71 patients with early-stage disease (Scheuer stages 1 and 2), NCPF was present in 20 (28.2%). Table 1 summarizes the characteristics of PBC patients with and without NCPF. Key findings revealed that the PBC + NCPF group was significantly older (54.2 ± 8.5 years vs. 49.4 ± 11.4 years, *p* = 0.04) and exhibited higher hepatocellular injury markers, including ALT (102.4 ± 88.9 U/L vs. 69.3 ± 47.1 U/L) and AST (103.2 ± 67.5 U/L vs. 70.3 ± 41.0 U/L; *p* = 0.02). The PBC + NCPF group also showed a more prominent increase in ALP (346.0 ± 226.9 U/L vs. 267.4 ± 199.3 U/L) and IgG (21.1 ± 9.3 g/L vs. 17.7 ± 6.2 g/L) levels; relatively higher proportion of women (96.7% vs. 90.3%), ANA positivity (80.0% vs. 62.9%), and AMA positivity (72% vs. 61.1%), although these differences were not statistically significant (*p* > 0.05). The overall proportion of splenomegaly was 42.4% in this study, with no significant differences between the two groups.

**Table 1:** Clinical and serological characteristics of patients, overall and stratified by the presence of NCPF.

Variable	All patients (*n* = 92)	PBC (*n* = 62)	PBC + NCPF (*n* = 30)	*p*-value
Age	51.0 ± 10.7	49.4 ± 11.4	54.2 ± 8.5	0.04
Women (%)	92.4%	90.3%	96.7%	0.28
ALT	80.2 ± 65.3	69.3 ± 47.1	102.4 ± 88.9	0.02
AST	80.9 ± 53.0	70.3 ± 41.0	103.2 ± 67.5	0.02
ALP	292.7 ± 210.6	267.4 ± 199.3	346.0 ± 226.9	0.10
GGT	302.0 ± 289.2	293.5 ± 297.8	320.5 ± 273.9	0.69
TBil	31.3 ± 34.0	27.5 ± 28.4	39.1 ± 42.8	0.20
DBil	17.8 ± 23.7	14.6 ± 18.8	23.4 ± 29.8	0.16
IgG	18.9 ± 7.5	17.7 ± 6.2	21.1 ± 9.3	0.12
IgM	4.6 ± 3.6	4.1 ± 2.5	5.5 ± 4.9	0.17
ANA+(%)	68.5%	62.9%	80.0%	0.15
AMA+(%)	64.4%	61.1%	72%	0.35
Splenomegaly	42.4%	41.9%	43.3%	0.899

ALT, alanine aminotransferase; AST, aspartate aminotransferase; ALP, alkaline phosphatase; GGT, γ-glutamyl transferase; TBil, total bilirubin; DBil, direct bilirubin; AMA, antimitochondrial antibody; ANA, antinuclear antibody; PBC, primary biliary cholangitis; NCPF, non-cirrhotic portal fibrosis.

Table 2 presents the prevalence of splenomegaly and NCPF stratified by Scheuer stages. Splenomegaly prevalence did not differ significantly across Scheuer stages, with 40% in stage 1, 37.9% in stage 2, and 57.1% in stage 3 (*p* = 0.179). NCPF demonstrated a progressive increase from stage 1 (20%) to stage 3 (47.6%), with intermediate prevalence in stage 2 (28.8%); however, this trend was not statistically significant (*p* = 0.092).

**Table 2:** Presence of splenomegaly and non-cirrhotic portal fibrosis (NCPF) across different Scheuer stages.

Feature	Stage 1 (*n* = 5)	Stage 2 (*n* = 66)	Stage 3 (*n* = 21)	*p*-value
Splenomegaly	40% (2)	37.9% (25)	57.1% (12)	0.179
NCPF	20% (1)	28.8% (19)	47.6% (10)	0.092

### Histological findings

**Figure 2:**
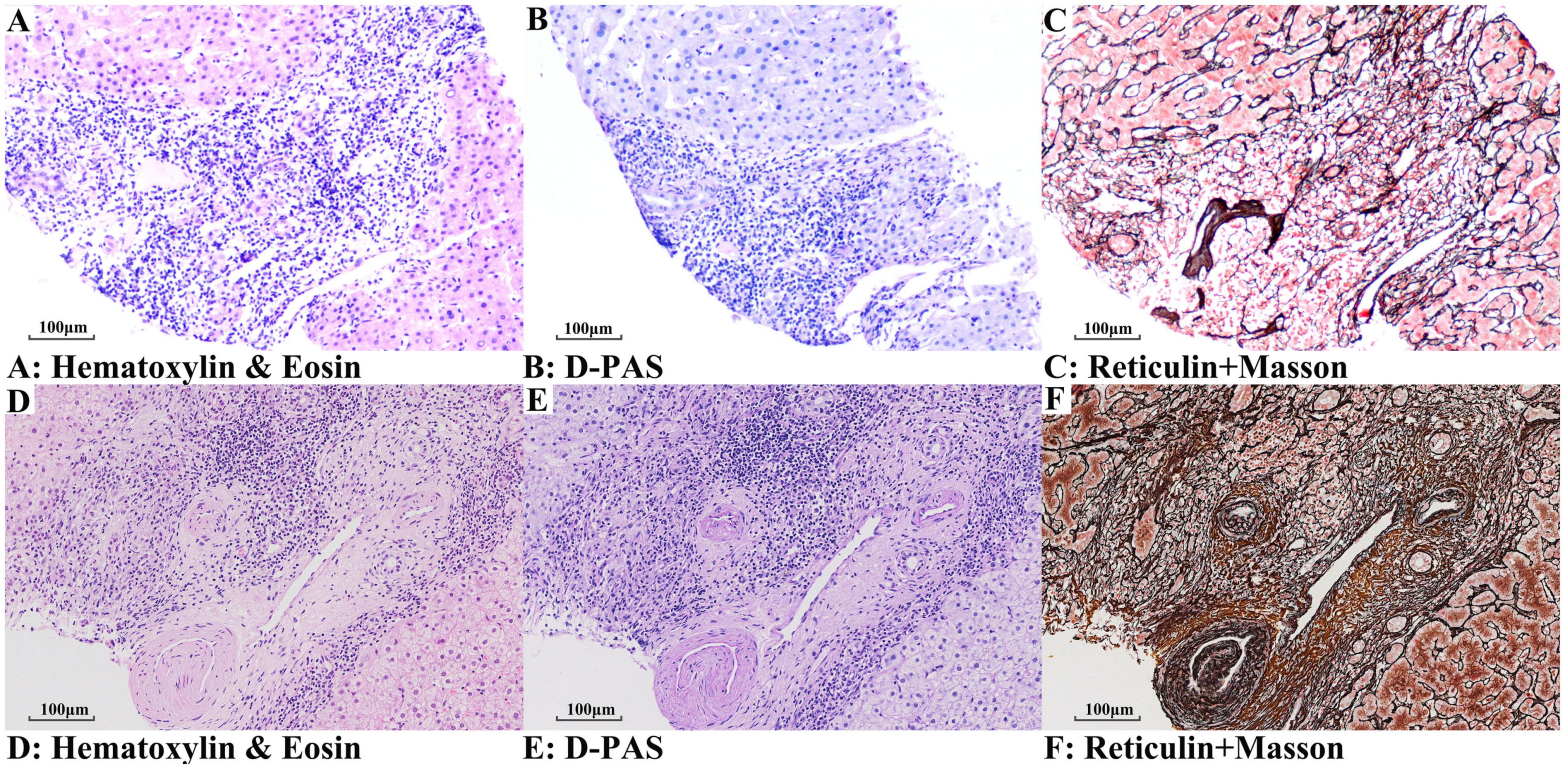
Contrasts on stage-2 PBC specimens, with and without concomitant NCPF. Panels **(A–C)** (PBC-only): **(A,B)** Small-caliber bile ducts encircled by dense lymphoplasmacytic infiltrates; and **(C)** Mild portal fibrosis, indicative of early-stage fibrosis associated with PBC. Panels **(D–F)** (PBC + NCPF): **(D,E)** Beyond the characteristic cholangitic lesions, portal veins exhibit pronounced luminal narrowing and wall sclerosis; and **(F)** Significant portal fibrosis, with fibrosis thickening of the portal vein wall (**A,D**: Hematoxylin and eosin staining; **B,E**: D-PAS staining; **C,F**: Reticulin + Masson staining).

**Figure 3:**
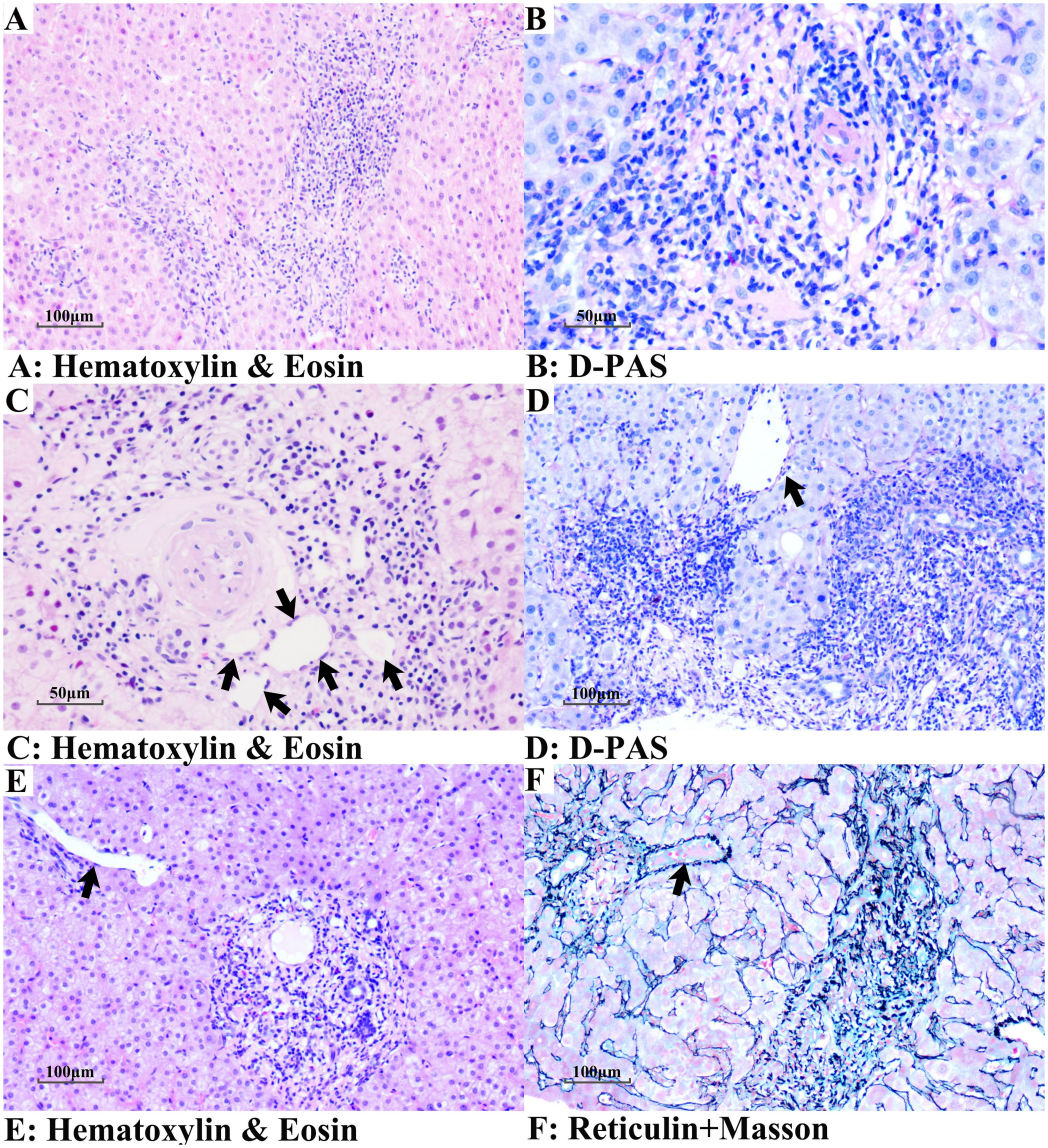
Aberrant microvessels in PBC + NCPF patients. **(A,B)** Disappearance of portal veins in portal areas. **(C)** Multiple thin-walled vascular structures, resembling angiomatous transformation (arrows). **(D,E)** Herniated portal veins (arrows). **(F)** Herniated portal vein in the upper left (arrow), loss of portal vein in the portal area on the right, and portal fibrosis (**A,C,E**: Hematoxylin and eosin staining; **B,D**: D-PAS staining; **F**: Reticulin + Masson staining).

The histological features of PBC (Scheuer stage 2) with and without NCPF are shown in Figure 2. Chronic non-suppurative destructive cholangitis was a hallmark histological feature of PBC (Figures 2A–C), with degenerative small-caliber bile ducts surrounded by dense lymphoplasmacytic infiltrates. In addition to typical histological changes of PBC, patients with PBC + NCPF showed features consistent with OPV (Figures 2D–F), including luminal narrowing, sclerosis, or disappearance of the portal vein branches. Portal fibrosis was more evident in the PBC + NCPF group compared to the PBC-only group (Figures 2C,F, 3F).

Aberrant vessels, such as a herniated portal vein and periportal angiomatosis, were also observed in PBC + NCPF patients (Figure 3). No cases of nodular regenerative hyperplasia (NRH) or incomplete septal cirrhosis (ISC) were found in this study.

**Figure 4:**
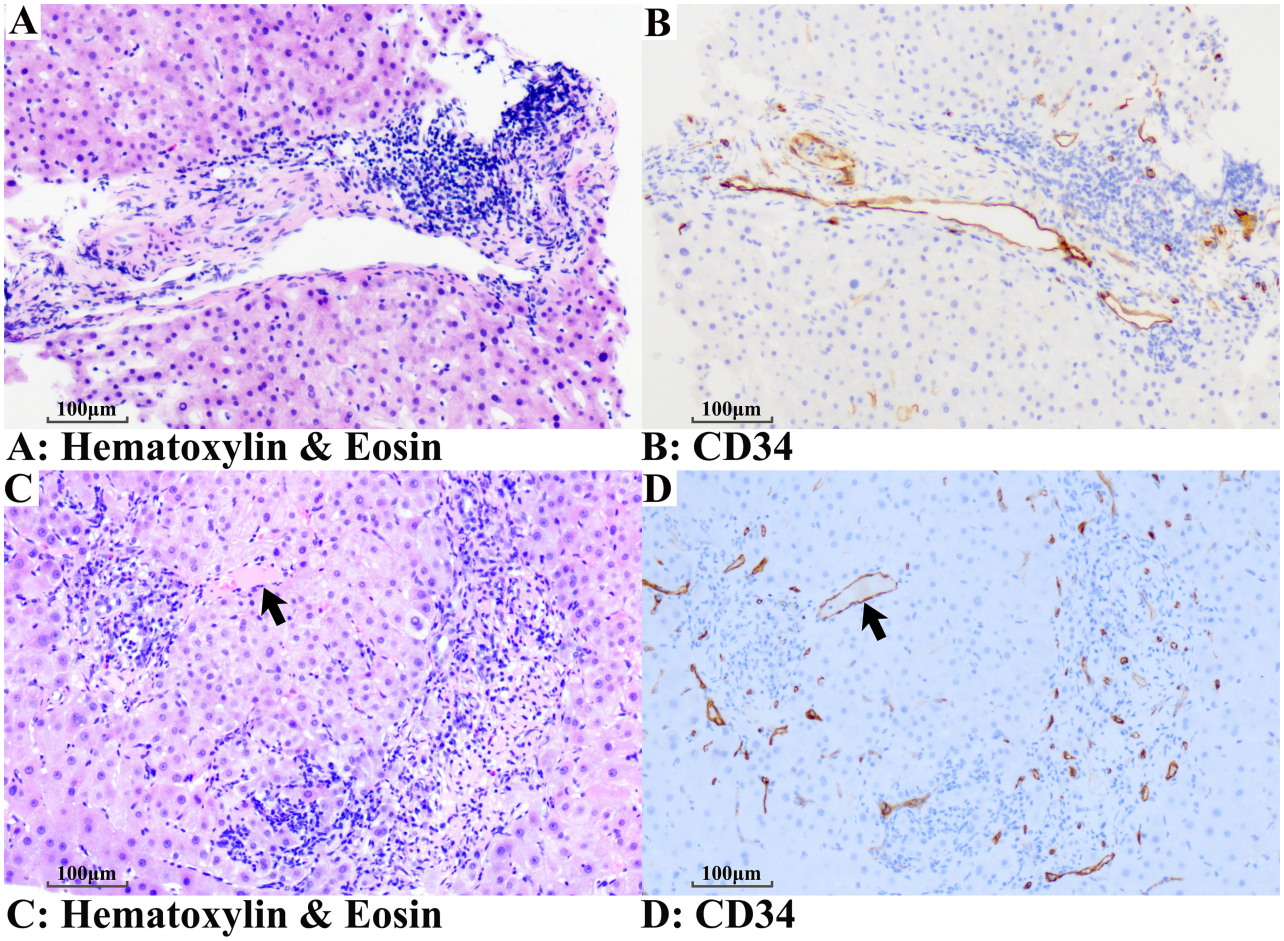
Microvascular alterations of patients with PBC + NCPF **(C,D)** in comparison with PBC-only **(A,B)**. **(A)** Portal lymphoplasmacytic infiltrates with a normal-sized portal vein. **(B)** Normal-sized portal vein accompanied by only a few microvessels. **(C)** Portal lymphoplasmacytic infiltrates and herniated portal vein (arrow). **(D)** Loss of normal-sized portal vein, herniated portal vein (arrow), and a notable proliferation of microvessels (**A,C**: Hematoxylin and Eosin staining; **B,D**: CD34 + staining).

Immunohistochemistry for CD34 was available only in a small subset of patients from both groups; therefore, these observations should be interpreted as exploratory and hypothesis-generating. Within this subset (five PBC-only vs. five PBC + NCPF), CD34 expression appeared more prominent in the PBC + NCPF group than in the PBC-only group, suggesting a potential association with microvascular remodeling in patients with non-cirrhotic portal fibrosis (Figure 4).

## Discussion

A substantial proportion of portal hypertension develops in the early stages of PBC, long before the development of cirrhosis (4–6). This study investigates the relationship between PBC and NCPF, a condition characterized by portal hypertension in the absence of cirrhosis. The emerging classification of NCPF within the broader spectrum of PSVD indicates a potential role of microvascular disturbances in NCPF in the development of portal hypertension in early PBC. This study identifies a previously unreported high prevalence of NCPF in pre-cirrhotic PBC (32.6% overall; 28.2% in early-stage disease), suggesting NCPF is substantially underrecognized in early PBC. The presence of NCPF is associated with higher biochemical activity and specific vascular histopathological alterations. Leveraging 15 years of tertiary-center data and APASL histopathological criteria, this study characterizes a clinicopathological cohort of NCPF-PBC overlap, proposing this entity as a model for investigating immune-mediated vascular injury within the spectrum of PSVD.

In this study, the PBC + NCPF group demonstrated increased ALT and AST and trends toward higher ALP and IgG, suggesting more active liver injury. Histologically, obliterative portal venopathy in the PBC + NCPF group was characterized by the disappearance or luminal narrowing of portal vein branches and aberrant vessels, aligning with well-described features of NCPF (15, 16) and consistent with the newly proposed PSVD criteria (8, 9). Furthermore, the PBC + NCPF group showed substantial alterations in portal vein architecture, with a marked elevation in microvessel count compared to the PBC-only group, implying an augmented angiogenic response. Another important finding was that PBC + NCPF had relatively higher proportions of ANA and AMA seropositivity. Increased angiogenesis and lymphoplasmacytic infiltration in PBC + NCPF highlight potential immune-driven vascular remodeling, reinforcing the link between autoimmunity and microvascular injury emphasized in rheumatological studies (10). Collectively, these findings position NCPF as a critical modulator of disease heterogeneity in early PBC, potentially indicating a more aggressive phenotype.

OPV was the only pathological pattern observed in NCPF cases in this study. The absence of NRH and ISC may indicate an early NCPF or PSVD phenotype in this cohort. Moreover, OPV is often considered more likely to arise secondary to biliary injury. According to Shan et al. (17), OPV demonstrated a significantly higher prevalence in both patients with biliary cirrhosis and corresponding rat models compared to those with hepatitis B-related cirrhosis. OPV acts as a pre-sinusoidal resistance factor, critically contributing to portal hypertension in biliary cirrhosis through bile duct proliferation-induced vascular compression (17). Notably, the elevated ALT and AST levels and OPV features (luminal narrowing and sclerosis) in the PBC + NCPF subgroup are consistent with Büyük et al. (18), who linked analogous vascular changes to idiopathic non-cirrhotic portal hypertension through CD34 + angiogenesis. Preliminary immunohistochemistry in this study suggested enhanced angiogenesis in PBC + NCPF (Figure 4), though larger validation studies are needed.

The findings of increased CD34 + microvessel density, lymphoplasmacytic infiltration, and elevated ANA/AMA seropositivity support interconnected vascular and immune dysregulation in the pathogenesis of PBC + NCPF. In previous studies evaluating the clinical features of NCPF, a high prevalence of autoantibodies and excessive lymphoproliferation was observed (19, 20). Moreover, recent advances have documented the efficacy of B-cell–targeting therapy in PBC, supporting the role of autoimmunity (21). This study proposes that chronic biliary inflammation may trigger sinusoidal remodeling and aberrant angiogenesis, fostering a pro-fibrotic microenvironment. This parallels the findings of De Gottardi et al., who characterized PSVD as a distinct entity involving endothelial injury and inflammatory cascades (8, 9). Lymphoplasmacytic infiltration adjacent to injured portal veins (Figures 2D–F) and elevated autoantibodies collectively suggest a potential immune component in microvascular injury, which is consistent with Tonutti et al., who emphasized autoimmune epithelitis in PSVD pathogenesis (10). Mechanistically, PBC-associated cholangitis may initiate periportal inflammation, culminating in secondary portal venopathy and microvascular thrombosis. Although the debate on the nomenclature of PSVD and NCPF continues (10, 22), recognizing immune-mediated vascular injury as a therapeutically targetable process in PBC remains important. Despite the exclusion of PBC from the current PSVD criteria (8), the high prevalence of microvascular alterations in PBC positions PBC + NCPF as a valuable model for investigating immune-mediated vascular injury within the PSVD spectrum. Further studies should explore whether NCPF–PBC overlap predicts accelerated disease progression or unique therapeutic implications.

The high NCPF prevalence (28%) in early PBC aligns with emerging evidence implicating the role of PSVD in non-cirrhotic portal hypertension among autoimmune liver diseases (4–6). This challenges the existing practice, which reserves vascular assessment for cirrhotic stages (1, 2). Incorporating NCPF screening into PBC biopsy protocols is supported by the results of this study, as it identifies patients with higher transaminases and active microvascular injury despite mild fibrosis. Notably, splenomegaly prevalence did not differ between the groups, underscoring the inadequacy of imaging alone for NCPF detection.

This study had several limitations. First, the sample size may limit the statistical power, particularly for subgroup analyses, and may not fully capture the heterogeneity of PBC and NCPF in broader populations. Second, the retrospective design inherently limits the ability to establish causal relationships and may introduce patient selection bias. Third, all clinical and pathological data were obtained during a specialist pathology consultation; therefore, longitudinal patient follow-up was not available. Fourth, non-invasive fibrosis assessment data (e.g., transient elastography/FibroScan) were not available because of the consultation-based retrospective design and the long study period, which began in 2006. Accordingly, this study could not assess non-invasive fibrosis measures or stiffness-histology correlations. Finally, immunohistochemical analyses (CD34) were available only for a small subset of patients in each group, limiting statistical power and generalizability. Future prospective studies with larger cohorts and robust control measures are essential to validate these findings and elucidate the clinical implications of PBC and NCPF.

In summary, this study identifies NCPF as a frequent, clinically significant feature in pre-cirrhotic PBC, characterized by distinct histopathological and serological signatures. Its recognition enables refined PBC subclassification and implicates immune-mediated microvascular injury as a tractable therapeutic target. Implementation of standardized NCPF screening in early PBC may optimize risk stratification, personalize management strategies, and ultimately mitigate portal hypertensive complications.

## Data Availability

The original contributions presented in the study are included in the article/supplementary material, further inquiries can be directed to the corresponding authors.
